# Administration of growth factors promotes salisphere formation from irradiated parotid salivary glands

**DOI:** 10.1371/journal.pone.0193942

**Published:** 2018-03-28

**Authors:** Vicky T. Nguyen, Peter Dawson, Qionghui Zhang, Zoey Harris, Kirsten H. Limesand

**Affiliations:** 1 Department of Nutritional Sciences, University of Arizona, Tucson, Arizona, United States of America; 2 Department of Biomedical Engineering, University of Arizona, Tucson, Arizona, United States of America; National Institute of Dental and Craniofacial Research, UNITED STATES

## Abstract

Worldwide, 500,000 cases of head and neck cancer (HNC) are reported each year and the primary treatment for HNC is radiotherapy. Although the goal of radiotherapy is to target the tumor, secondary exposure occurs in surrounding normal tissues, such as the salivary glands. As a result, despite successful treatment of the cancer, patients are left with long-term side effects due to direct damage to the salivary glands. The effect is chronic and currently there is no treatment. Stem cells are an attractive therapeutic option for treatment of radiation-induced glandular dysfunction because of the potential to regenerate damaged cell populations and restore salivary gland function. However, limited knowledge about the endogenous stem cell population post irradiation hinders the development for stem cell-based therapies. In this study, an ex vivo sphere formation cell culture system was utilized to assess the self-renewal capacity of cells derived from parotid salivary glands at a chronic time point following radiation. Salivary glands from irradiated mice generate significantly fewer salispheres, but can be stimulated with fetal bovine serum (FBS) to generate an equivalent number of salispheres as unirradiated salivary glands. Interestingly, the number and size of salispheres formed is dependent on the concentration of FBS supplemented into the media. Salispheres derived from irradiated glands and cultured in FBS media were found to contain cells that proliferate and express progenitor and acinar cell markers such as Keratin 5, Keratin 14, Aquaporin 5, and NKCC1. Utilization of insulin-like growth factor (IGF1) injections following radiation treatment restores salivary gland function and improves salisphere generation. These findings indicate that stimulation of these cellular populations may provide a promising avenue for the development of cell-based therapies for radiation-induced salivary gland damage.

## Introduction

Annually, head and neck cancer (HNC) has more than 500,000 new cases worldwide [1]. The current treatment for HNC utilizes a multimodality approach, which includes surgery in combination with radiotherapy (RT) and chemotherapy [2]. Despite the application of intensity-modulated radiation therapy (IMRT) to spare the salivary glands during HNC treatment, the parotid glands are frequently still damaged due to close proximity to the tumor sites [3–5]. Consequently, HNC patients often suffer from permanent xerostomia following radiotherapy treatment [6]. Current preventive and palliative care for the management of RT-related xerostomia are limited and largely ineffective [7–9]. This poses a health concern because reductions in the quantity of saliva can greatly affect the quality of life of HNC patients. Such patients suffer from increased oral infection rates, difficulty chewing and swallowing food, malnutrition, and difficulty in speaking [10–11].

Adult stem and progenitor cells (SPCs) have been identified in many tissues (e.g. skin, gut, bone marrow) and are known to play an important role in the continuous repair and regeneration of tissues following damage [12–14]. In many tissues, stem cells are activated to initiate the repair process when there is an excessive loss of differentiated cells or by environmental cues. This is followed by the proliferation and differentiation of progenitor cells to replenish the existing cell pool [15–17]. In accordance with this paradigm of stem cell-based regeneration, it is thought that putative salivary stem/progenitor cells (SSCs) have the potential to regenerate salivary glands following radiation damage [18–19]. However, it has been demonstrated both clinically and in mouse models of radiation-induced salivary gland damage that there is a lack of regeneration following radiation treatment [20–21]. Ionizing radiation exposure can disrupt tissue homeostasis and regeneration by either 1) directly damaging the SPC pool or 2) indirectly disturb the *in vivo* environment that is necessary to maintain the stem/progenitor pools [22–23]. It remains elusive whether the lack of regeneration by salivary glands following radiation treatment is due to direct damage to the SSC pool or disruption of the SSC niche environment.

Previous studies have shown that transplantation of putative salivary stem/progenitor cells, based on identification of cell surface markers c-Kit^+^, CD24^+^, Lineage^-^, Sca-1^+^, one day following radiation treatment can partially restore salivary gland function [24–26]. Additionally, administration of growth factors (e.g. Insulin-like growth factor 1 (IGF1), Keratinocyte growth factor (KGF), Glial cell line-derived neutrophic factor (GDNF)) at an early critical time point (1–4 days) following radiation treatment have also been effective in restoring salivary gland function [26–28]. Taken together, these findings suggest that at an early time point following radiation treatment, the putative SSC pools are not lost in the course of radiation and are still responsive to external stimuli. However, it remains unclear whether salivary stem/progenitor cells retain their regenerative capacity when stimulated by an external cue at chronic time points post radiation damage. It would provide great therapeutic insight to understand the “window of opportunity” where these cellular populations could be stimulated to regenerate during chronic time points of radiation-induced salivary gland dysfunction.

Sphere-forming assays have been widely used in other systems, including the brain, lung, pancreas, and breast to retrospectively characterize a heterogeneous population of cells that exhibit stem cell-like behavior based on the capacity to self-renew and differentiate *in vitro* [29]. In this study, we utilized an ex vivo sphere formation cell culture to assess the self-renewal capacity of cells derived from parotid glands at a chronic time point following radiation treatment. There was a significant reduction in sphere formation from irradiated parotid glands and supplementation with growth factors restored self-renewal properties to levels similar to unirradiated controls. Defining the self-renewal and differentiation potential of irradiated salivary cells may provide therapeutic value for the development of regenerative therapies for damaged salivary glands.

## Materials and methods

### Mice and sphere forming assay

All experiments were conducted in 4–6 week old or 8 week old female FVB mice. All mice were maintained and treated in accordance with protocols approved by the University of Arizona Institutional Animal Care and Use Committee (IACUC). A total of 44 mice were used for sphere culture across 22 individual experiments. For each experiment, parotid glands from 2 mice were collected and salisphere formation was propagated using a sphere culture technique as previously described [24, 30]. Cells were suspended in serum free medium supplemented with or without 2.5%, 5%, or 10% fetal bovine serum (FBS) (Hyclone, Logan, UT) and plated at a density of 200,00 cells in ultra-low attachment plates (Corning, Corning, NY). Culture condition and numbers of primary cell preparations for each experiment are indicated in each figure legend.

### Radiation treatment

Mice were anesthetized with Ketamine/Xylazine (50mg/kg (K)+10 mg/kg (X)) followed by exposure to a single dose of 5 Gy targeted to the head and neck region using a ^60^Cobalt Teletherapy unit (Theratron-80, Atomic Energy of Canada Ltd), with the rest of the body protected by a lead shield. The 5 Gy dose was chosen based on previous work demonstrating significant reductions in salivary function and differentiation status (as determined by amylase expression) of parotid salivary glands [27]. Thirty days after irradiation, a sub-group of irradiated mice were injected intravenously through the tail vein with 5μg recombinant IGF-1 (GroPrep, Adelaide, Australia) for three consecutive days (days 31–33, approximately 24 hours apart). This dose of IGF-1 was chosen because it was previously shown to restore salivary gland function post-radiation treatment [27].

### Saliva collection

Eight week old female FVB mice were treated with targeted head and neck radiation with and without post-therapy IGF1. On day 60 after radiation treatment, mice were injected i.p. with carbachol (0.25 mg/kg body weight) and saliva was collected for 5 minutes. Total saliva was collected by vacuum aspiration from 6 mice per treatment group: untreated FVB, irradiated, and IGF1 treated group on ice following carbachol injection into pre-weighted tubes and stored at -80°C as previously described [31–32].

### Cell viability assay

Cell viability was assessed using the thiazolyl blue tetrazolum bromide (MTT; Thermo Scientific, Waltham, MA) assay. At pre-determined time points, the MTT solution (5mg/mL) was added to each well for 4 hours at 37°C. Formazan crystals formed in salisphere cells were solubilized with DMSO (Fisher Scientific, Waltham, MA). Lysates were transferred to 96-well plates (Corning) and absorbance was measured at 540 nm using the Ultramark Microplate Reader (Biorad, CA, USA).

### EdU incorporation and immunofluorescence staining

At pre-determined time points, salispheres were fixed directly in culture by adding 1 volume of 10% buffered formalin for 20 minutes at room temperature. After fixation, salispheres were permeabilized with 0.2% Triton X in PBS for 20 minutes at room temperature. Target-specific staining of salispheres was performed in suspension by incubating in primary antibody diluted 1:100 in 1% BSA overnight at 4°C: anti-Ki-67 (12201, Cell Signaling, Danvers, MA), anti-Keratin 5 (PRB-160P, Covance, Princeton, NJ), anti-Keratin 14 (PRB-155P, Covance), anti-Aquaporin 5 (AB3559, Labome, Princeton, NJ), and anti-NKCC1 (a59791, Abcam, Cambridge, UK); followed by incubation in secondary anti-rabbit Alexa 594 (A-11307, Invitrogen, Carlsbad, CA) at 1:500 dilution. For EdU incorporation, primary salispheres were incubated in culture medium containing 10μM EdU (5-ethynyl-2′-deoxyuridine, Invitrogen) for one hour prior to fixation. The EdU staining protocol was adapted from manufacturer’s instructions (Click-It Plus EdU Alexa Fluor 488 Imaging Kit, Life Technologies, Grand Island, NY) to stain salispheres directly in suspension. Salispheres were pelleted by centrifugation at 4000 rpm for 5 minutes and resuspended in EdU click-it cocktail for 30 minutes at room temperature. Images were acquired with an Intelligent Imaging Innovations (Danvers, Colorado) configured instrument including a Zeiss Marianas 100 Microscopy Workstation (Oberkochen, Germany), Yokogawa CSU-X1 M1 Spinning Disk (Musashino, Tokyo, Japan) and Photometrics Evolve 512 EMCCD (Tucson, Arizona) using a Zeiss EC Plan-Neifluar 40X/1.3 NA oil objective and a .5μm z-separation between the slices in the stack. The Spinning Disk includes a Semrock Di01-T488/568 dichroic beam splitter and a Em01-R488/568 dual bandpass emission filter for 488 nm ex- BP 525/43 em and 561 nm ex- BP 642/117 em imaging.

### Western blot analysis

Salispheres were collected, rinsed with phosphate-buffered saline (PBS), and lysed in RIPA buffer that contained 5 mM sodium orthovanadate (Fisher Scientific) and protease inhibitor cocktail (Sigma-Aldrich, St. Louis, MO) on ice for 30 minutes. Supernatants were harvested after centrifugation at 12,000 rpm for 15 minutes (4°C). Five micrograms of protein sample was loaded onto 10% polyacrylamide gels and then transferred to 0.45 μm Immobolin-P membranes (Millipore, Billerica, MA). The membranes were blocked with 5% BSA and then immunoblotted overnight at 4°C with one of the following antibodies: anti-Keratin 5, anti-Keratin 14, and anti-ERK 1/2 (1:2000, Cell Signaling). For detection, SuperSignal substrate was used as instructed by the manufacturer (Thermo Scientific).

### Determination of size and total number of salispheres

Salisphere number and size were assessed on Days 4–14 in culture. To do this, 25 bright field images were randomly captured from each well using Optixcam Summit OSC-3.0x TMS-DK3-5 camera (Microscope Store, Roanoke, VA, USA). The diameter of individual salispheres was measured using 1.46 ImageJ software. For each experiment, the number of salispheres was manually counted from a minimum of 15 images per well from 3 to 10 wells. The number of wells analyzed for salisphere count for each experiment is indicated in each figure legend. Only salispheres with a diameter of >50μm were counted due to the utilization of a 40 μm diameter filter to obtain a single cell suspension prior to plating.

### Proliferation analysis

Proliferation of salispheres was assessed through Ki-67 immunostaining on different days of culture. To do this, between 10–20 confocal 3D stack images per treatment condition were randomly selected for analysis. The number of 3D stack images used for the proliferation analysis in each experiment is indicated in each figure legend. Cells were manually counted from every fifth slice in the z-stack image using the Cell Counter program from ImageJ. Proliferation of an individual salisphere was determined by the sum of the number of positive cells/total number of cells from the accumulated slices in a single z-stack image. Analysis of EdU, Keratin 14, and Keratin 5 levels were carried out using a similar manual counting technique.

### Statistical analyses

Statistical analyses were performed using SPSS (version 24.0, SPSS Inc., Chicago, IL, USA). Two-sided independent sample t-tests were performed to compare the number of salispheres between treatment groups at different days in culture. One-way ANOVA was performed on the log transformed data of Ki-67 proliferation index of an individual salisphere to satisfy the normality assumption. Tukey post hoc multiple comparisons were conducted to compare the differences in salisphere proliferation between groups of different salisphere sizes, days in culture, and treatment conditions. One-way ANOVA was performed on dual expression of EdU/Keratin 14 and EdU/Keratin 5 by salisphere cells. No transformation was needed to satisfy the assumptions of normality and homogeneity of variances. Tukey post hoc multiple comparisons were conducted to compare differences in EdU/Keratin 14 and EdU/Keratin 5 dual expression between groups of culture conditions and treatment conditions. Similarly, one-way ANOVA with Tukey post hoc comparisons were run to compare mean salivary flow rates across untreated control, irradiated, and IGF1 treated groups. Graphical generation was done using Graph-Pad (version 5.0, San Diego, CA).

## Results

### Sphere-forming efficiency decreases following radiation

**Fig 1:**
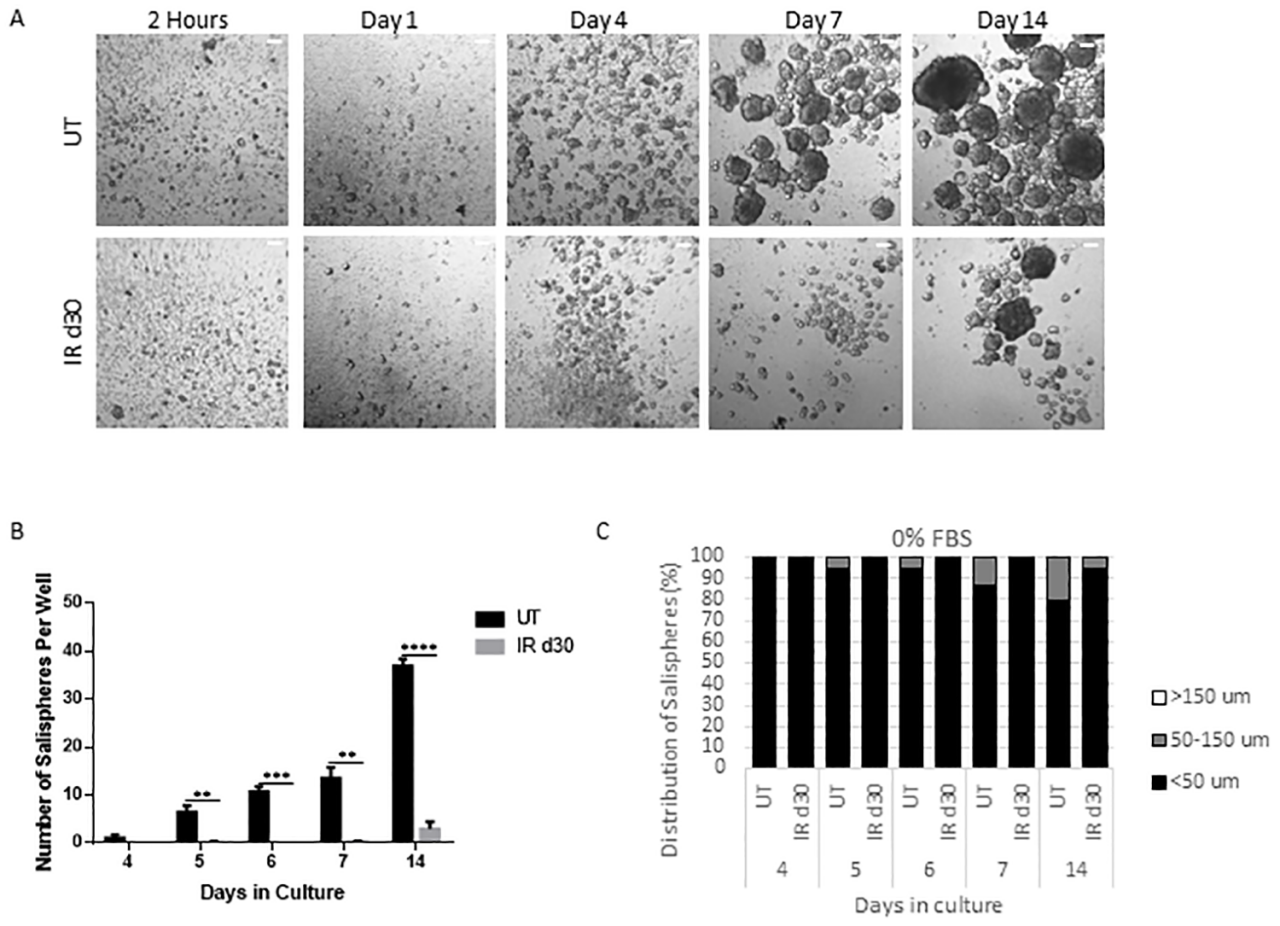
Sphere-forming efficiency decreases following radiation. A single 5 Gy dose of radiation (IR d30) was given to 4–6 week old female FVB mice and parotid glands were collected 30 days following radiation for sphere formation assay. Representative bright field images of salispheres grown from untreated control (UT) and irradiated (IR d30) glands in serum-free media at different time points in culture (A). Number of salispheres was quantified per 6 wells from 3 primary cell preparations per treatment group at days 4, 5, 6, 7, and 14 in culture and expressed as average ± SEM (B). Representative graphs showing the distribution of salisphere sizes among all the salispheres counted from one UT and IR d30 primary sphere preparation (C). P values were obtained with 2-sided unpaired t-test (n = 3), **p < .01, ***p < .001, ****p < .0001. Scale bar = 50μm.

**Fig 2:**
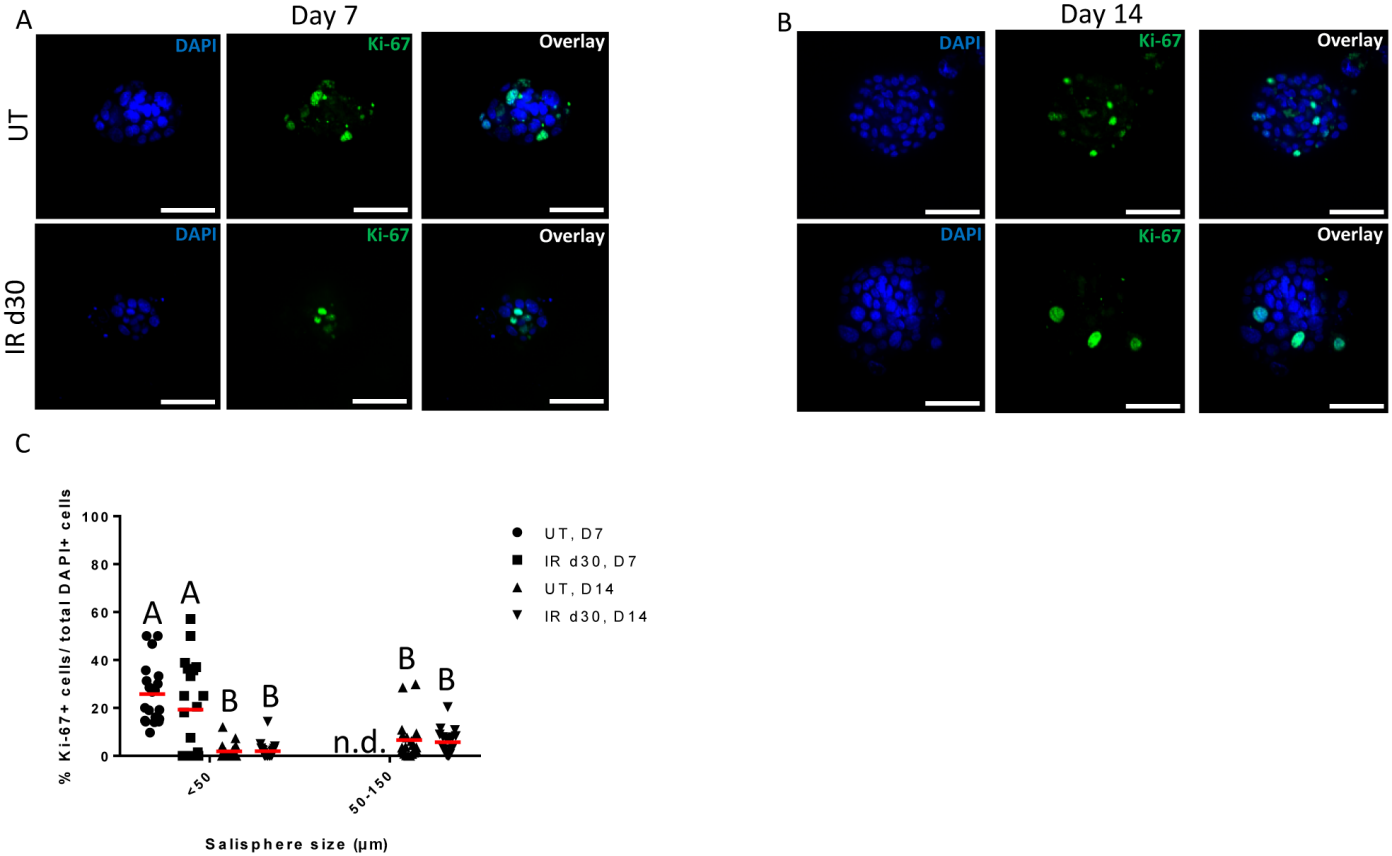
Similar proliferation rates are observed in untreated and irradiated salispheres cultured in serum-free conditions. Untreated and irradiated parotid-derived salispheres were fixed after 7 and 14 days in culture and stained for Ki-67 (green). Representative confocal immunofluorescence images are shown. Ki-67 was expressed in salisphere-derived cells at both early (day 7) and later (day 14) time points in culture (A-B). Percentage of Ki-67+ proliferating cells was quantified from 20 salispheres, of different sizes (<50μm, 50–150μm), at day 7 and 14 for both treatment groups and expressed as percent of proliferation by individual salisphere (red bar = mean percent of proliferation by group). At day 7 in culture, medium-sized salispheres (50–150μm) were rarely detected, thus this analysis was not determined (n.d.). Significant differences (p< 0.05) were determined using a one-way ANOVA with post-hoc Tukey multiple-comparison tests on the log transformed data of Fig 2C. Groups with the same letter are not significant different from each other (C). Scale bar = 50μm.

To evaluate the sphere-forming efficiency of irradiated parotid glands, the number and size of salispheres were quantified over time in culture. FVB mice (4–6 week old) were subjected to a single 5 Gy dose of targeted head and neck radiation and parotid glands were harvested 30 days post treatment for sphere-forming assay. On each cell isolation day, spheres were grown from unirradiated mice for comparison. By day 7, the presence of salispheres can be detected and the number and size of salispheres increased with longer time in culture (Fig 1A). Assessment of salisphere formation reveals a significant decrease in the number of salispheres formed in the irradiated glands compared to untreated controls (Fig 1B). MTT (3-(4,5-dimethylthiazol-2-yl)-2,5-diphenyltetrazolium bromide) assay did not detect a difference in cell viability between cells derived from untreated and irradiated glands under serum-free medium condition (S1 Fig). The proliferation capacity of untreated and radiation-derived salispheres was also assessed through Ki-67 immunostaining. At early time points (days 4–7), the culture consists mostly of free-floating clusters of cells that are less than 50μm in diameter, which will be referred to as small salispheres (Figs 1C, 2A and 2B). The proliferation percentage of small salispheres ranges from 10–50% in the untreated group and 0–60% in the irradiated group (Fig 2C). Interestingly ~35% of the spheres in the irradiated group exhibit no proliferation at day 7. Due to the different responses to growth in the irradiated spheres, there is no significant difference in proliferation compared to unirradiated controls (P = 0.078). Following a longer period of time in culture (day 14), the proliferation capacity of salispheres derived from untreated and irradiated cells significantly decreases, regardless of salisphere size (Fig 2C). These results suggest that decreased salisphere formation from irradiated glands may not be due to a decrease in cell viability, rather a subset population that is less responsive to proliferate in serum-free medium condition at early time points in culture.

### FBS increases sphere-forming efficiency of irradiated parotid-derived cells

**Fig 3:**
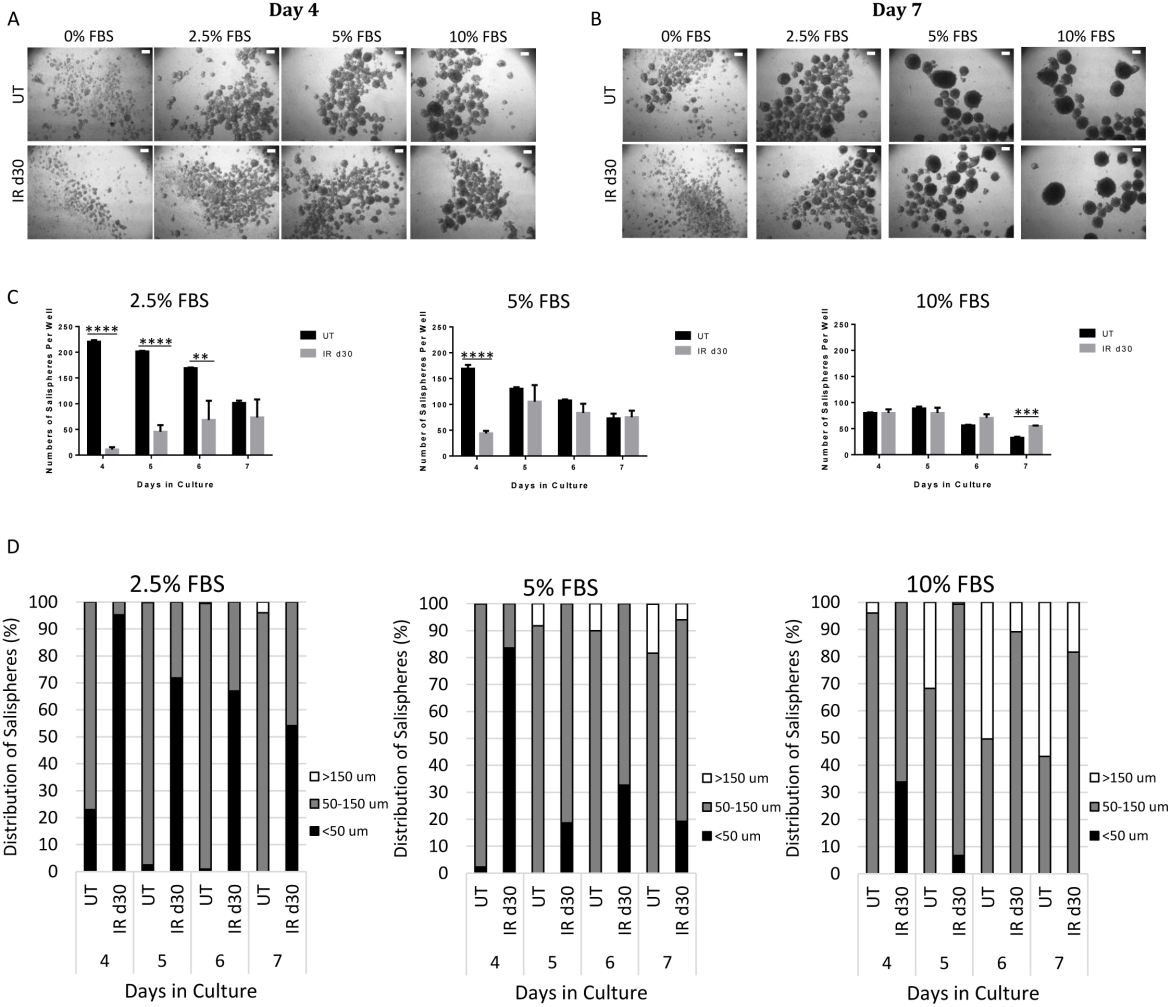
FBS increases sphere-forming efficiency of irradiated parotid-derived cells. Representative bright field images of salispheres grown from UT (untreated) and IR d30 (irradiated) glands in serum-free media, supplemented with different concentrations of fetal bovine serum (FBS), taken at day 4 (A) and 7 (B) in culture. Number of salispheres was quantified per 6 wells from 3 primary cell preparations per treatment group at days 4–7 in culture and expressed as average ± SEM. P values were obtained with a 2-sided unpaired t-test (n = 3), **p < .01, ***p < .001, ****p < .0001 (C). Representative graphs showing the distribution of salisphere sizes among all the salispheres counted from one UT and IR d30 primary sphere preparation (D). Scale bar = 100μm.

To determine whether sphere-forming cells from irradiated mice can be stimulated to respond and form salispheres, fetal bovine serum (FBS) was supplemented into the culture medium and the number and size of salispheres were determined at 4, 5, 6, and 7 days after the start of culture (Fig 3). By day 4, the presence of salispheres can be detected in both untreated and irradiated groups. Increasing FBS concentration and longer time in culture results in the formation of larger salispheres (Fig 3A and 3B). For the untreated group, the total number of salispheres in 2.5%, 5%, and 10% FBS with a diameter (D_s_) >50 μm gradually decreases with longer time in culture. By 7 days in culture, the number of salispheres falls to a level of approximately one-half of the initial value (2.5% FBS: 221 at day 4 vs. 102 at day 7; 5% FBS: 169 at day 4 vs. 73 at day 7; 10%: 80 at day 4 vs. 33 at day 7). In contrast, the total number of salispheres from irradiated mice gradually increases with 2.5% and 5% FBS culture conditions (2.5% FBS: 10 at day 4 vs. 73 at day 7; 5% FBS: 44 at day 4 vs. 75 at day 7). At days 4, 5, and 6 in culture, the total number of 2.5% FBS-treated salispheres derived from irradiated glands is significantly lower than the number of salispheres from unirradiated mice. In addition, at day 4 in culture, the total number of irradiated 5% FBS-treated salispheres is also significantly lower compared to untreated group. In contrast, there is no difference in the number of salispheres formed between untreated and irradiated groups when supplemented with a higher concentration of FBS (10%) (Fig 3C).

In parallel, as the total number of salispheres fell, both the maximal diameter (D_s_^max^) and the proportion of larger salispheres increases (Fig 3D). At day 7 in culture, almost all the 2.5% FBS-treated salispheres from the untreated group have a diameter value between 50 and 150 μm. In the untreated group, large spheres (D_S_ >150μm) emerge at an earlier time in culture (day 5) with supplementation of higher FBS concentration (5% and 10%), and their proportion increases until the end of the culture period (day 7). Compared to the untreated group, the D_s_^max^ of FBS-treated salispheres derived from irradiated glands is smaller and within a size range between 50 and 150 μm. Even with longer time in culture and the addition of higher FBS concentrations, the proportion of large-size salispheres (D_S_ >150μm) is less than untreated group (Fig 3D).

It was previously reported that there is nearly a 2-fold decrease in the number of salispheres formed from the submandibular glands harvested from old mice compared to young mice [33]. Since the majority of HNC patients are adults and 4–6 week old mice could be defined as juveniles, we evaluated whether mice that were 8 weeks old at the time of radiation exhibited a similar phenotype when salisphere cultures were evaluated 30 days after radiation treatment. Similar to what was previously observed (Fig 2), FBS supplementation of irradiated cells derived from mice that were 8 weeks old at the time of treatment, increases the number of salispheres along with the formation of larger salispheres upon longer culture periods (S2 Fig). These results suggest that the addition of FBS improves salisphere formation from irradiated parotid glands, albeit the rate at which salispheres are formed and enlarged is lower compared to untreated controls.

### Proliferation rates are similar in salispheres supplemented with FBS from untreated and irradiated mice

**Fig 4:**
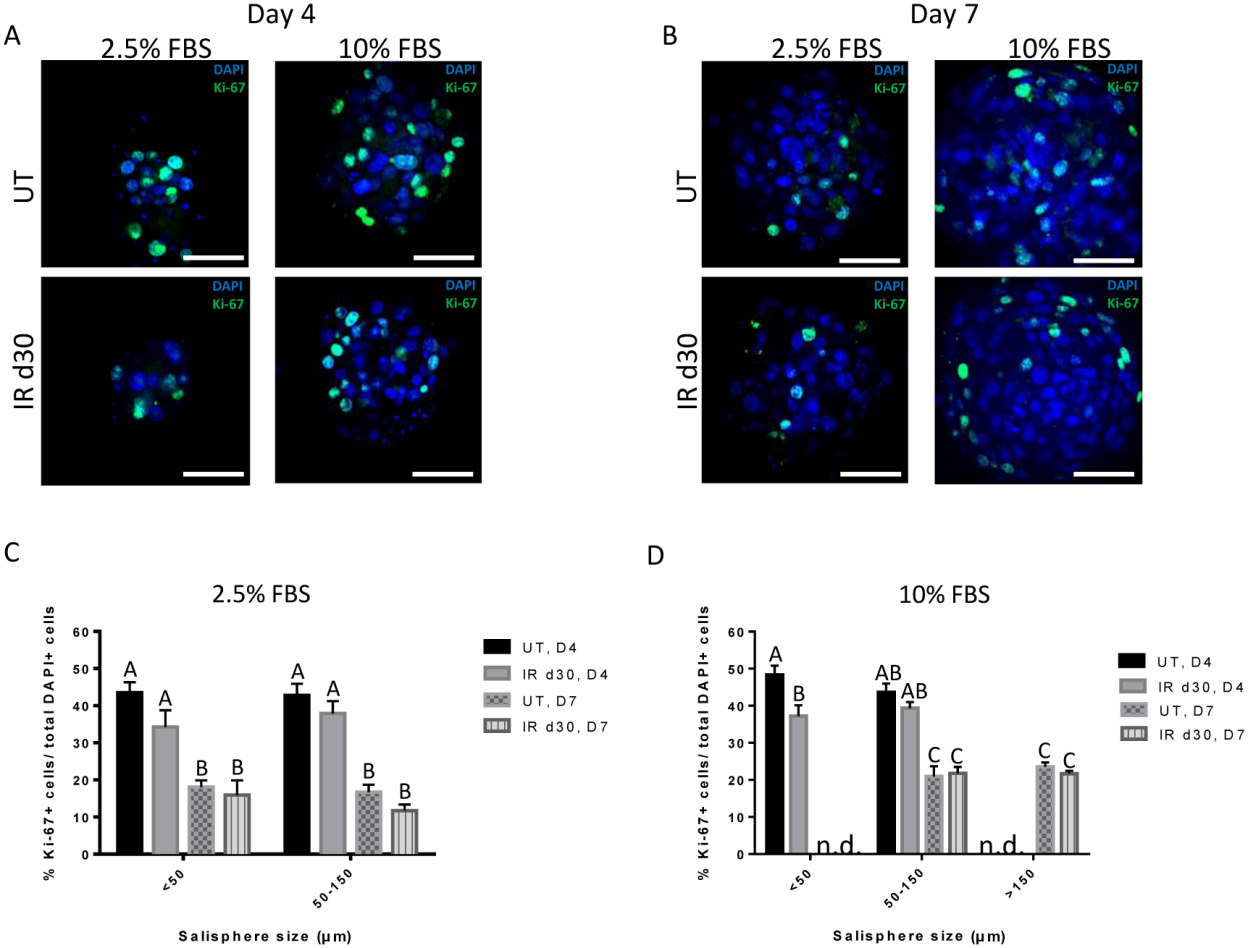
Similar proliferation rates are observed in untreated and irradiated salisphere cultures supplemented with FBS. Untreated and irradiated parotid-derived salispheres, maintained under different FBS concentrations, were fixed after 4 and 7 days in culture and stained for Ki-67 (green). Representative confocal immunofluorescence images are shown (A-B). Percentage of Ki-67+ proliferating cells was quantified from 20 salispheres, of different sizes (<50μm, 50–150μm, >150μm) and maintained under different FBS concentration (2.5% and 10%), at days 4 and 7 for both treatment groups and expressed as average ± SEM. At day 4 in 10% FBS culture condition, large-sized salispheres (>150μm) were rarely detected. Likewise, small-sized salispheres (<50μm) were rarely observed at day 7 in culture. Thus, these analyses were not determined (n.d.). Significant differences (p< 0.05) were determined using a one-way ANOVA followed by post-hoc Tukey multiple-comparison tests on the log transformed data of Fig 4C and 4D. Groups with the same letter are not significant different from each other. (C-D). Scale bar = 50μm.

To investigate whether the increase in sphere-forming efficiency of irradiated parotid-derived cells is due to an increase in the proliferative capacity in the presence of FBS, salispheres supplemented with 2.5% or 10% FBS were fixed after 4 and 7 days in culture and immunostained for Ki-67 (Fig 4A and 4B). There is an increase in the mean proliferation percentage of 2.5% FBS-treated salispheres (Fig 4C and 4D) derived from untreated glands compared to salispheres maintained in serum-free culture (0–50μm: 44.95% at day 4 in 2.5% FBS vs. 27.27% at day 7 in 0% FBS). Regardless of salisphere size, there is no significant difference in proliferative capacity of salispheres derived from untreated glands maintained in 2.5% FBS (Day 4: 44.58% in small salispheres (D_S_ <50μm) vs. 43.87% in medium sized salispheres (D_S_: 50–150μm)). However, the rate of proliferation of 2.5% FBS-treated salispheres derived from untreated glands significantly decreases with longer time in culture (small salisphere (D_S_ <50μm): 44.58% at day 4 vs. 18.46% at day 7; medium salispheres (D_S_: 50–150μm): 40.63% at day 4 vs. 15.97% at day 7). Overall, it appears that the proliferation rate of salispheres from untreated and irradiated mice is similar when considering salisphere size, different FBS concentrations (2.5% vs. 10%) or days in culture (Fig 4C and 4D). Salispheres derived from mice that were 8 weeks old at the time of treatment, proliferate in culture with a similar pattern with regards to salisphere size, FBS supplementation and time in culture (S3 Fig). These results indicate that FBS supplementation stimulates proliferation rates of salispheres to a similar extent in untreated and irradiated salivary glands and increasing time in culture decreases total proliferation rates.

### Expression of salivary stem/progenitor and acinar cell markers in salispheres

**Fig 5:**
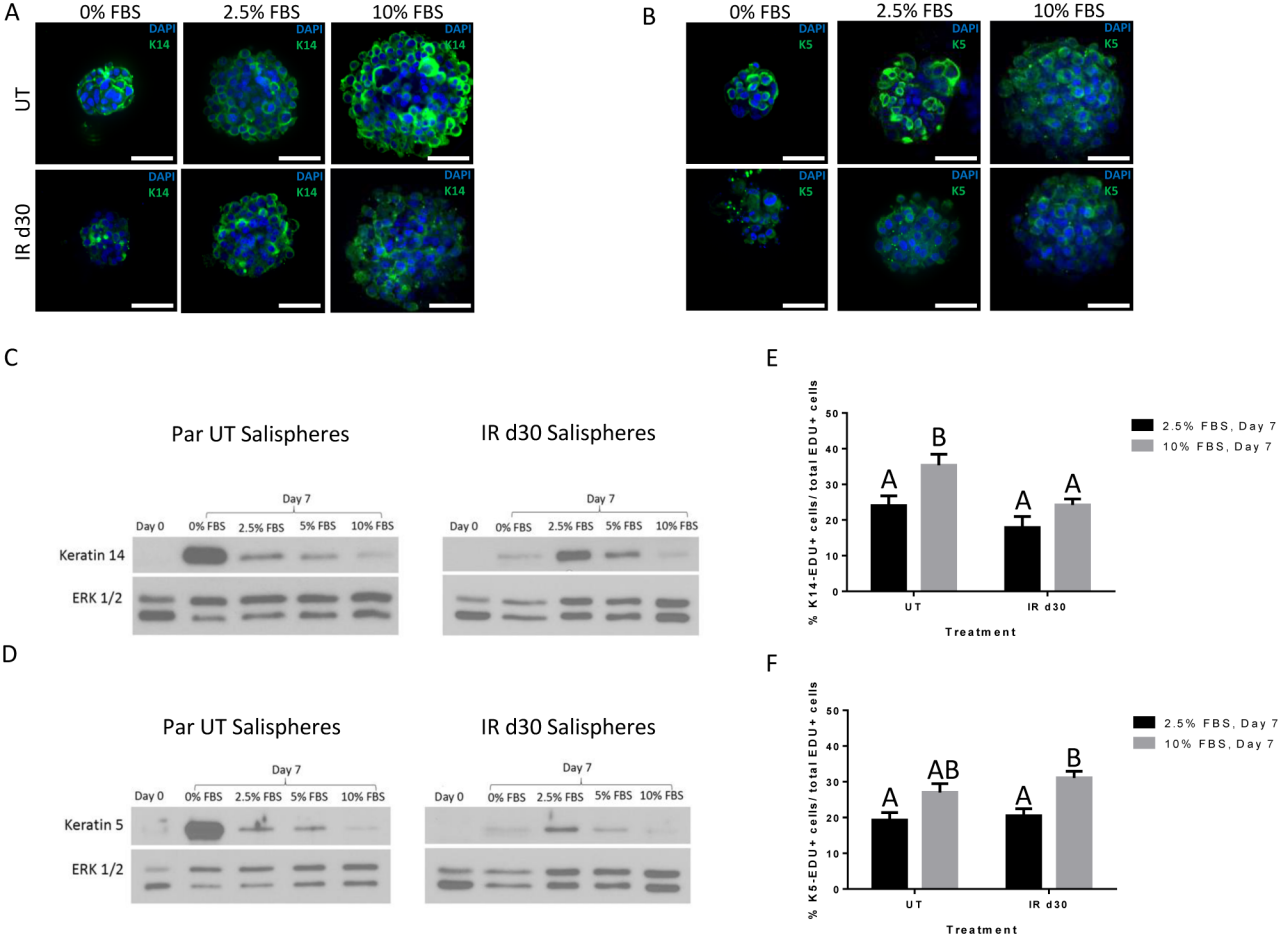
Expression of salivary stem/progenitor markers by salisphere cells. Untreated and irradiated parotid-derived salispheres, maintained under different FBS concentrations, were fixed after 7 days in culture and stained for Keratin 5 (K5) or Keratin 14 (K14) (green). Representative confocal immunofluorescence images are shown (A-B). Representative immunoblot of protein extracts from untreated and irradiated salispheres grown under different FBS concentration medium, collected at day 7 in culture, and probed for Keratin 5 and Keratin 14 (C-D). On day 7 of culture, salispheres were labelled with EdU for 1 hour *in vitro*, fixed, and dual stained for EdU and Keratin 5 or Keratin 14. Percentage of dual EdU-K14+ or Edu-K5+ cells was quantified from 20 salispheres, maintained under different FBS concentrations, for both treatment groups and expressed as mean percent of K14+ or K5+ proliferating cells (± SEM) within an individual salisphere. Significant differences (p< 0.05) were determined using a one-way ANOVA followed by post-hoc Tukey multiple-comparison tests. Groups with the same letter are not significant different from each other (E-F). Scale bar = 50μm.

Previous studies have reported that salispheres are highly enriched in Keratin 5 (K5)- and Keratin 14 (K14)-positive cells, two progenitor markers that are known to be important during embryonic development of submandibular glands (SMG) [26, 34–35]. To determine if salispheres derived from untreated and irradiated parotid glands maintained under different FBS concentrations express the aforementioned markers, we performed immunofluorescence staining following 7 days in culture (Fig 5A). Keratin 5 and 14 positive cells are observed in salispheres derived from both untreated and irradiated parotid glands grown in each FBS culture condition. The presence of K5 and K14 in FBS-treated salispheres was confirmed with immunoblots (Fig 5C and 5D). The highest total levels of K14 or K5 are detected in 0% FBS conditions for salispheres from untreated mice versus 2.5% FBS conditions for salispheres from irradiated mice. To investigate the contribution of Keratin 5- and Keratin 14-positive cells to the increased proliferation observed with FBS supplementation, salispheres were incubated with EdU one hour prior to collection and dual immunostained for EdU and K14 or K5. Quantification of EdU/K14 dual positive cells reveals that 24% and 18% of the proliferating population of cells expressed the progenitor marker K14 from untreated and irradiated salispheres maintained in 2.5% FBS respectively. With regard to the expression profile of Keratin 5 under 2.5% FBS medium condition, 19.6% and 21.7% of the total EdU+ cells in salispheres derived from untreated and irradiated parotid glands express Keratin 5. A slightly higher contribution of K14 and K5 cells to the total proliferative population is observed in salispheres maintained in 10% FBS and this is statistically significant for EdU/K14 dual positive cells derived from untreated mice and EdU/K5 dual positive cells derived from irradiated mice. When directly comparing salisphere cells derived from untreated and irradiated glands, no significant differences are observed in the percentage of EdU/K14+ and EdU/K5+ cells.

Additionally, it was recently shown that salispheres contained cells with acinar cell characteristics, as indicated by the increase in expression of acinar cell markers aquaporin-5 (Aqp5) and NKCC1 [36]. To assess the acinar cell phenotype in sphere culture, salispheres derived from both untreated and irradiated parotid glands, grown in serum-free (0% FBS) and FBS supplemented (2.5% FBS) culture conditions, were stained with Aqp5 and NKCC1. Aqp5- and NKCC1-expressing cells are observed in both FBS treated and non-FBS treated salispheres (S4 Fig). Taken together, these data suggest that K14, K5, Aqp5, and NKCC1 continue to be expressed in salisphere cultures supplemented with FBS and contribute to the overall proliferative rate of salispheres.

### In vivo post-radiation administration of IGF1 increases sphere-forming efficiency of irradiated parotid-derived cells

**Fig 6:**
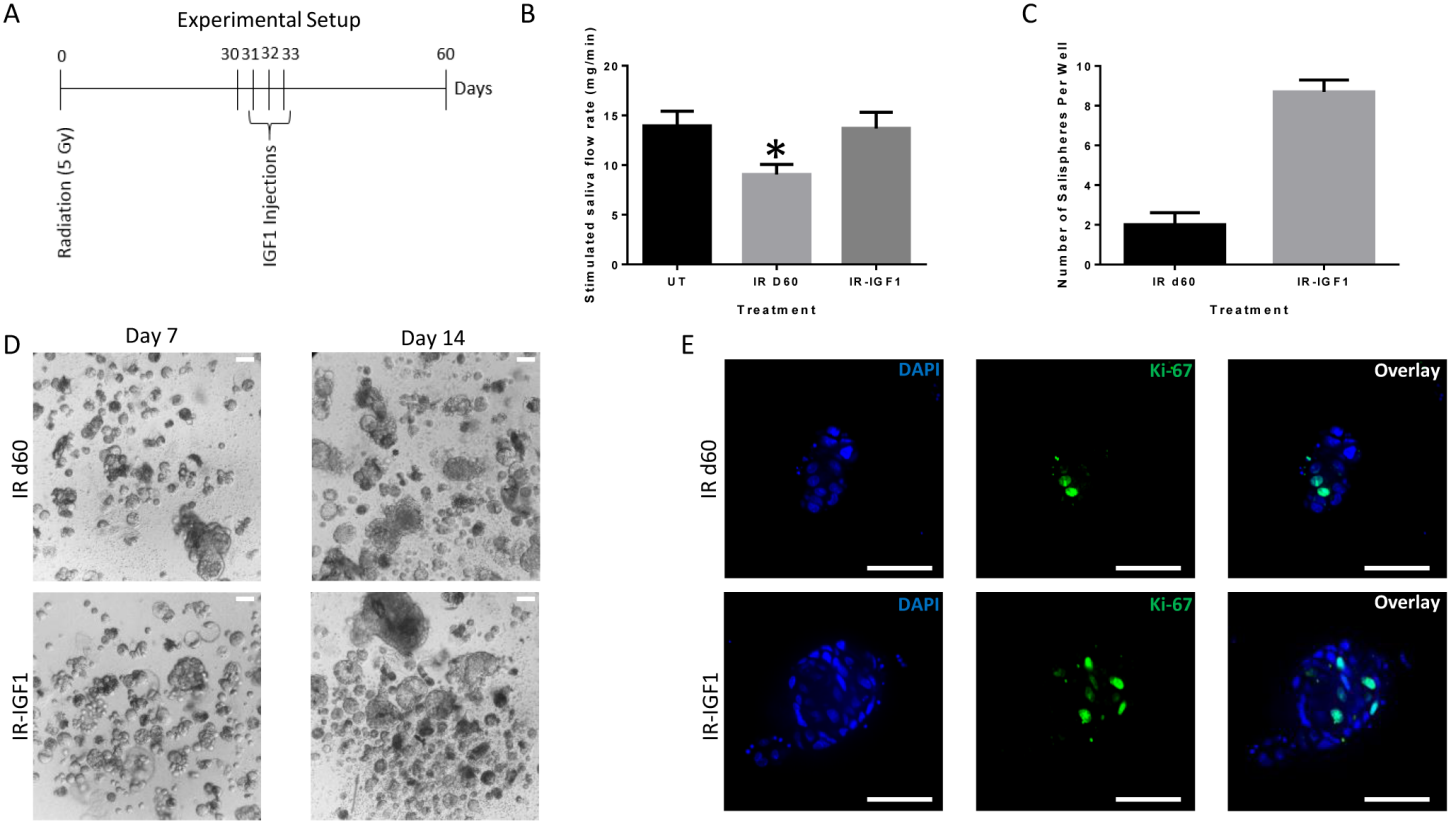
Post-radiation treatment with IGF1 increases sphere-forming efficiency of irradiated parotid-derived cells. A single 5 Gy dose of radiation was given to 4–6 week old or 8 week female FVB mice followed by injections of insulin growth factor 1 (IGF1) on days 31–33 as depicted (A). Stimulated salivary flow rates were determined as described in the materials and methods section on day 60. The graph represents the mean saliva flow rate from 6 mice (8 week old) per treatment group: untreated control (UT), irradiated (IR d60), and IGF1 treated (IR-IGF1). Significant differences (*p<0.05) were determined using a one-way ANOVA followed by a post-hoc Tukey multiple comparison test (B). Thirty days following IGF1 treatment, parotid glands were collected for sphere formation assay. Representative graph of the average number (± SEM) of salispheres from 10 wells per treatment group on day 7 in culture from one IR d60 and IR+IGF primary sphere preparation (C). Representative bright field images of salispheres grown from irradiated (IR d60) and IGF1 treated (IR-IGF1) glands in serum-free media at different time points in culture (D). Irradiated (IR d60) and IGF1 (IR-IGF1) treated parotid-derived salispheres were fixed after 7 days in culture and stained for Ki-67 (green). Representative confocal immunofluorescence images are shown (E). Scale bar = 50μm.

Previous work from our lab has demonstrated that administration of IGF1 four days after radiation leads to full restoration of salivary function [27]. The post-therapeutic effect of IGF1 on salivary function following radiation treatment led us to hypothesize that IGF1, like FBS, may increase the sphere-forming efficiency of irradiated parotid-derived cells. To this end, 4–6 week old FVB mice were subjected to a single dose of 5 Gy radiation and thirty days following radiation treatment, IGF1 was administered (Fig 6A). At day 60, salivary flow rates are restored in IGF1 treated mice (Fig 6B) and parotid glands were collected for sphere-forming assay. There is an increased number of salispheres formed in the IGF1-treated glands when compared to irradiated glands (Fig 6C). Ki-67, Aqp5, and NKCC1 immunofluorescent staining of salispheres shows that IGF1 salisphere-derived cells are proliferating and exhibit acinar cell markers under serum-free culture conditions (Fig 6D and S5 Fig). Similar effects on salisphere generation were observed when 8 week old mice were subjected to the same experimental design (S6 Fig). These data suggest that following radiation treatment, a cellular population remains that can be stimulated *in vivo* with IGF1 to restore sphere-forming capacity thirty days later.

## Discussion

Stem cell therapy holds great promise for the treatment of salivary gland dysfunction, the most common post-therapy complication for head and neck cancer patients [24–26, 37]. Previous studies have shown that expansion of the SSC pool either prior to radiation treatment or one day following radiation exposure through administration of growth factors (e.g. KGF, GDNF) partially restored salivary gland function [26, 28]. This suggested that at an early acute phase (one day post radiation), the SSC pool is not depleted and can be triggered to respond given the right stimuli. Recently, it was shown that the percentage of salivary gland label-retaining cells, which resembled SSCs in terms of *in vitro* self-renewal capacity and expression of stem/progenitor markers, is maintained as late as 30 days following radiation treatment [38]. However, it is not completely understood whether at chronic time points following radiation damage, putative SSCs can still be stimulated and involved in repair and regeneration of salivary glands. The current study demonstrates that a subset population of parotid-derived cells capable of self-renewal in ex vivo sphere-forming assay are still present 30 days after radiation and respond to administration of extracellular growth factors.

In accordance with previous findings in the submandibular gland [28, 39], our data demonstrates that a single dose (5 Gy) of radiation results in a significant reduction in the number of salispheres formed in culture from parotid glands. Interestingly, the decrease in sphere formation does not appear to be due to a loss in cell viability. Quantification of Ki-67 levels shows that 35% of salispheres derived from irradiated glands exhibit zero Ki-67 positive cells after seven days in serum-free medium culture. In contrast, all salispheres from untreated glands contained Ki-67 positive cells. This suggests that radiation could induce a non-responsive state in SSC; however due to the considerable variability in proliferation levels there were no significant differences between treatment groups. After 14 days in culture, the proliferative capacity is significantly reduced when compared to day 7 irrespective of radiation treatment. Previous work from other sphere culture systems (e.g. neurosphere, mammosphere) have demonstrated that prolonging the time period of primary culture significantly reduced the proliferation of sphere-forming cells [40–41]. This may suggest that the unresponsive phenotype observed in the irradiated parotid derived cells at an early time in culture (day 7) may not be due to the duration of the culture condition, but due to the inherent dormant state of the cells following radiation treatment.

It was previously reported by Tatsuishi et al. that salivary stem/progenitor cells were still present in submandibular glands surgically resected from patients who underwent radiotherapy, but the cells are in a dormant, quiescent state [42]. Findings from this study lead us to hypothesize that the decrease in salisphere formation may be a feature of quiescent irradiated SSCs. To determine whether supplementation with growth factors would stimulate cells derived from the irradiated parotid glands to proliferate and form salispheres in culture, we supplemented the serum-free media with FBS. Regardless of treatment, the addition of FBS to the medium stimulated the formation of large salispheres. Interestingly, compared to untreated controls, the size of salispheres derived from irradiated cells is smaller when cultured in a low concentration of FBS, suggesting a dose-response effect. However, while the rates at which salispheres are formed and enlarged are lower compared to untreated controls, there is no significant difference in proliferation rates of salispheres derived from untreated glands compared to irradiated glands, regardless of FBS concentration, salisphere size, or days in culture. Similar effects were observed when radiation was conducted on 8 week old mice. Our results demonstrate that the addition of FBS, which contains a cocktail of growth factors, promotes the expansion of salispheres from irradiated parotid glands. More importantly, we show that 30 days post treatment, a time point that continues to exhibit a significant loss of salivary function, the irradiated cells can be stimulated by growth factors *in vitro* and form spheres in culture. This suggests that even at a later time point following radiation, there is a subset population of cells that retain the capacity for self-renewal.

Genetic lineage tracing experiments in a SMG development model showed that Keratin 5+ (K5) and Keratin 14+ (K14) cells are multipotent progenitor cells that give rise to various cell types in the epithelial compartment of the submandibular glands [34–35, 43]. In the current study, immunohistochemical (IHC) staining and immunoblotting data demonstrate that salispheres derived from irradiated glands continue to express Keratin 5 and Keratin 14. Additionally, approximately 20–30% of proliferating cells are K5 or K14 positive within salispheres derived from irradiated glands. At day 7, both FBS and non- FBS treated salispheres contained Aqp5+ and NKCC1+ cells. This suggests that FBS supplementation did not seem to alter the cellular composition (SSC or acinar) within salispheres derived from untreated or irradiated mice.

We have previously shown that stimulation with IGF1 after radiation-induced salivary gland dysfunction has been initiated leads to a restoration of stimulated salivary flow rates [27]. The current study extended this model by delaying the IGF1 injections to day 30 instead of day 4 and determined salisphere formation thirty days later (radiation day 60). We observe that IGF1 stimulation *in vivo* can promote self-renewal capacity of the irradiated cells remaining at radiation day 30 when evaluated by an ex vivo sphere assay at radiation day 60. A recent study by Marmary et al. demonstrated that 60 days following radiation, there is a prominent increase in senescence-associated β galactosidase activity within the ductal cell compartment of irradiated SMG glands [44]. The authors postulated that senescence-mediated events contribute to the chronic loss of salivary gland function post irradiation. Our study suggests that 30 days post-radiation may be within a window of opportunity where the parotid salivary cells can still be stimulated and restoration of function is obtainable.

In summary, our study demonstrates that cells derived from irradiated parotid glands require an external stimulus to maximize ex vivo expansion. Characterization and stimulation of this salivary stem/progenitor cell sub-population may provide a promising avenue for cell-based therapy for radiation-induced salivary damage in a clinical setting.

## Supporting information

S1 Fig Cell viability assay of salispheres derived from untreated and irradiated mice. Viability of salisphere cells derived from untreated (UT) and irradiated (IR d30) parotid glands was assessed at different time points in serum-free culture using MTT assay. Absorbance measurement was obtained from one UT and IR d30 primary sphere preparation (A).
(TIF)

S2 Fig FBS increases sphere-forming efficiency of irradiated parotid derived cells from adult salivary glands. A single 5 Gy dose of radiation (IR d30) was given to 8 week old female FVB mice and parotid glands were collected 30 days following irradiation for sphere formation assay. Representative bright field images of salispheres grown from UT (untreated) and IR d30 (irradiated) glands in serum-free media, supplemented with different concentration of fetal bovine serum (FBS), taken at day 4 (A) and 7 (B) in culture. Representative graph of the average number (± SEM) of salispheres from 3 wells per treatment group days 4–7 in culture from one UT and IR d30 primary sphere preparation. Representative graphs showing the distribution of salisphere sizes among all the salispheres counted from one UT and IR d30 primary sphere preparation (D). Scale bar = 100μm.
(TIF)

S3 Fig Similar proliferation rates are observed in untreated and irradiated salisphere cultures from adult salivary glands. Untreated (UT) and irradiated (IR d30) parotid-derived salispheres from 8 week old female FVB mice, maintained under different FBS concentrations, were fixed after 4 and 7 days in culture and stained for Ki-67 (green). Representative confocal immunofluorescence images are shown (A-B). Percentage of Ki-67+ proliferating cells was quantified from 10 salispheres, of different sizes (<50μm, 50–150μm, >150μm) and maintained under different FBS concentration (2.5% and 10%), at day 4 and 7 for both treatment groups and expressed as average ± SEM (C-D). At day 4 in 10% FBS culture condition, large-sized salispheres (>150μm) were rarely detected. Likewise, small-sized salispheres (<50μm) were rarely observed at day 7 in culture. Thus these analyses were not determined (n.d.). Scale bar = 50μm.
(TIF)

S4 Fig Expression of acinar cell markers by salisphere cells derived from untreated and irradiated mice. Untreated (UT) and irradiated (IR d30) parotid-derived salispheres, maintained under different FBS concentrations, were fixed after 7 days in culture and stained for Aquaporin 5 (AQP5) and NKCC1 (green). Representative confocal immunofluorescence images are shown (A-B). Scale bar = 50μm.
(TIF)

S5 Fig Expression of acinar cell markers by salisphere cells derived from irradiated mice receiving post therapy IGF1. Salispheres grown from IGF1 treated parotid glands, maintained in serum free media, were fixed after 14 days in culture and stained for Aquaporin 5 (AQP5) and NKCC1 (green). Representative confocal immunofluorescence images are shown (A-B). Scale bar = 50μm.
(TIF)

S6 Fig Post-treatment of IGF1 increases sphere-forming efficiency of irradiated parotid-derived cells from adult salivary glands. A single 5 Gy dose of radiation was given to 8 week old FVB mice followed by injections of insulin growth factor 1 (IGF1) on days 31–33 as depicted in Fig 6A. Thirty days following IGF1 treatment, parotid glands were collected for sphere formation assay. Representative bright field images of salispheres grown from irradiated (IR d60) and IGF1 treated (IR-IGF1) glands in serum-free media at different time points in culture (A). Representative graph of the average number (± SEM) of salispheres from 10 wells per treatment group on day 7 in culture from one IR d60 and IR+IGF primary sphere preparation (B). Irradiated (IR d60) and IGF1 treated (IR-IGF1) parotid-derived salispheres were fixed after 7 days in culture and stained for Ki-67 (green). Representative confocal immunofluorescence images are shown (C). Scale bar = 50μm.
(TIF)
