# Supplementary figures and images for: Administration of growth factors promotes salisphere formation from irradiated parotid salivary glands
Source: PLoS One. 2018 Mar 28;13(3):e0193942. doi: 10.1371/journal.pone.0193942 (PMC5873995; doi:10.1371/journal.pone.0193942)

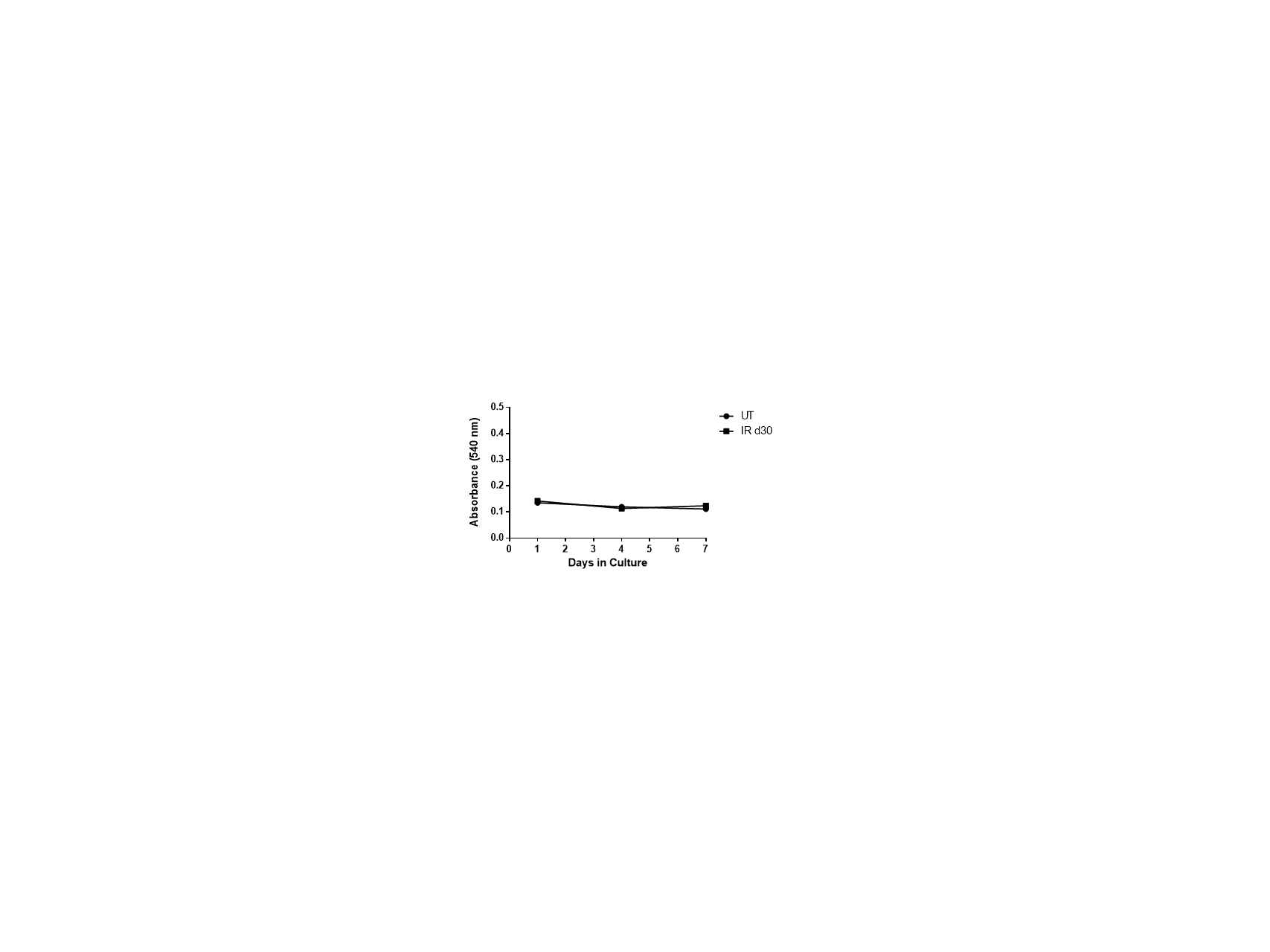

Supplement: S1 Fig — Viability of salisphere cells derived from untreated (UT) and irradiated (IR d30) parotid glands was assessed at different time points in serum-free culture using MTT assay. Absorbance measurement was obtained from one UT and IR d30 primary sphere preparation (A). (TIF) [file pone.0193942.s001.tif]

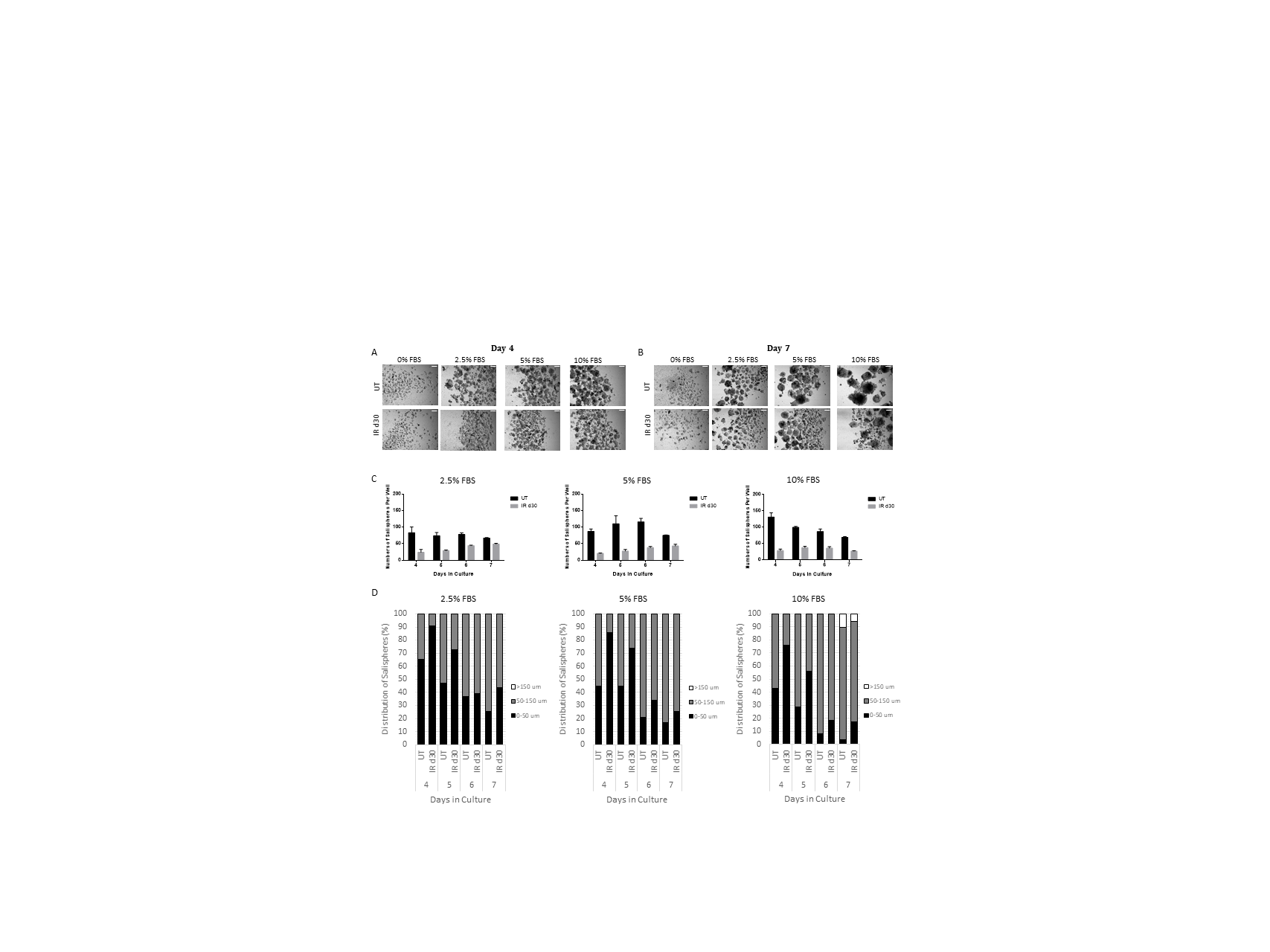

Supplement: S2 Fig — A single 5 Gy dose of radiation (IR d30) was given to 8 week old female FVB mice and parotid glands were collected 30 days following irradiation for sphere formation assay. Representative bright field images of salispheres grown from UT (untreated) and IR d30 (irradiated) glands in serum-free media, supplemented with different concentration of fetal bovine serum (FBS), taken at day 4 (A) and 7 (B) in culture. Representative graph of the average number (± SEM) of salispheres from 3 wells per treatment group days 4–7 in culture from one UT and IR d30 primary sphere preparation. Representative graphs showing the distribution of salisphere sizes among all the salispheres counted from one UT and IR d30 primary sphere preparation (D). Scale bar = 100μm. (TIF) [file pone.0193942.s002.tif]

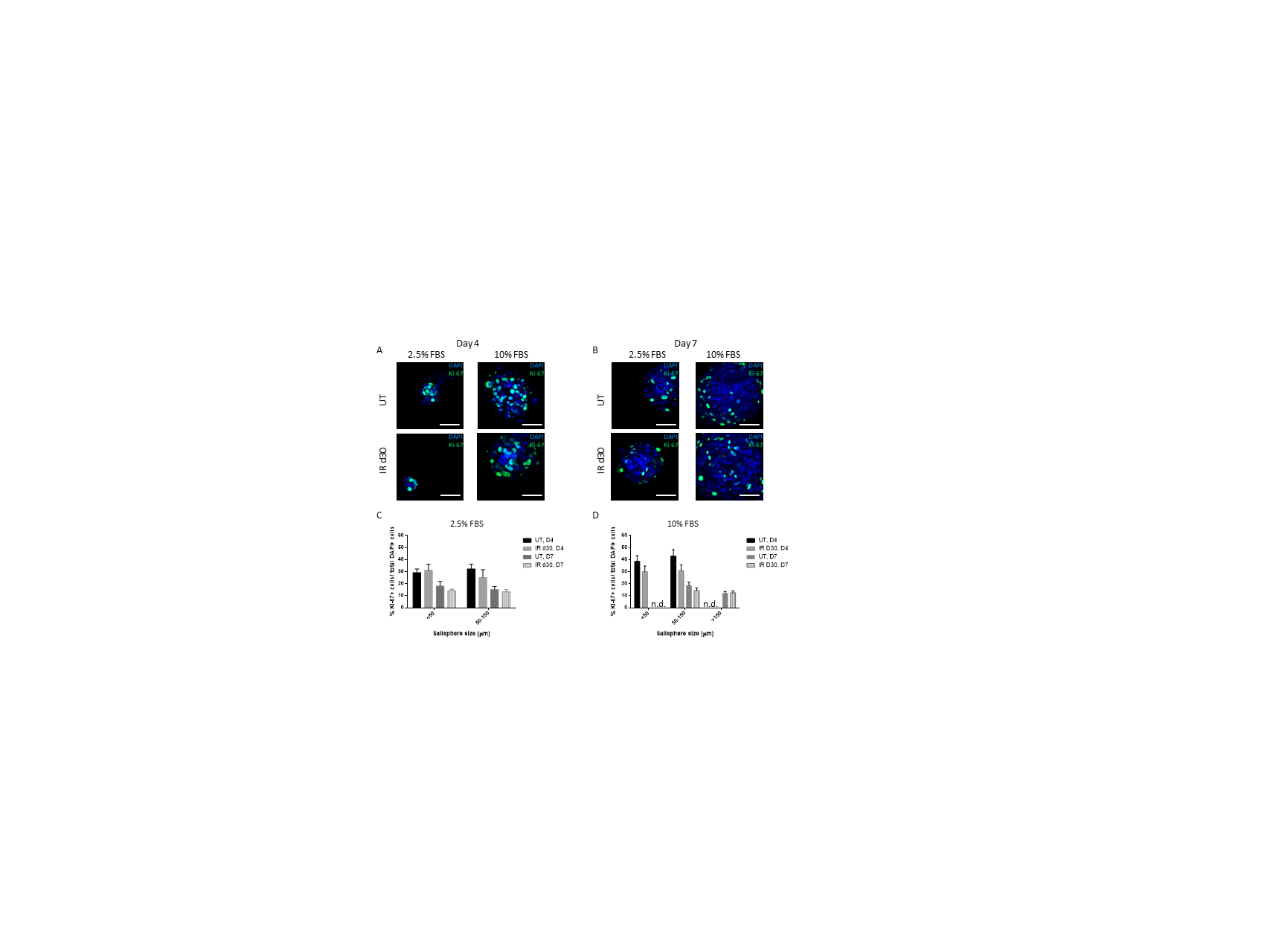

Supplement: S3 Fig — Untreated (UT) and irradiated (IR d30) parotid-derived salispheres from 8 week old female FVB mice, maintained under different FBS concentrations, were fixed after 4 and 7 days in culture and stained for Ki-67 (green). Representative confocal immunofluorescence images are shown (A-B). Percentage of Ki-67+ proliferating cells was quantified from 10 salispheres, of different sizes (<50μm, 50–150μm, >150μm) and maintained under different FBS concentration (2.5% and 10%), at day 4 and 7 for both treatment groups and expressed as average ± SEM (C-D). At day 4 in 10% FBS culture condition, large-sized salispheres (>150μm) were rarely detected. Likewise, small-sized salispheres (<50μm) were rarely observed at day 7 in culture. Thus these analyses were not determined (n.d.). Scale bar = 50μm. (TIF) [file pone.0193942.s003.tif]

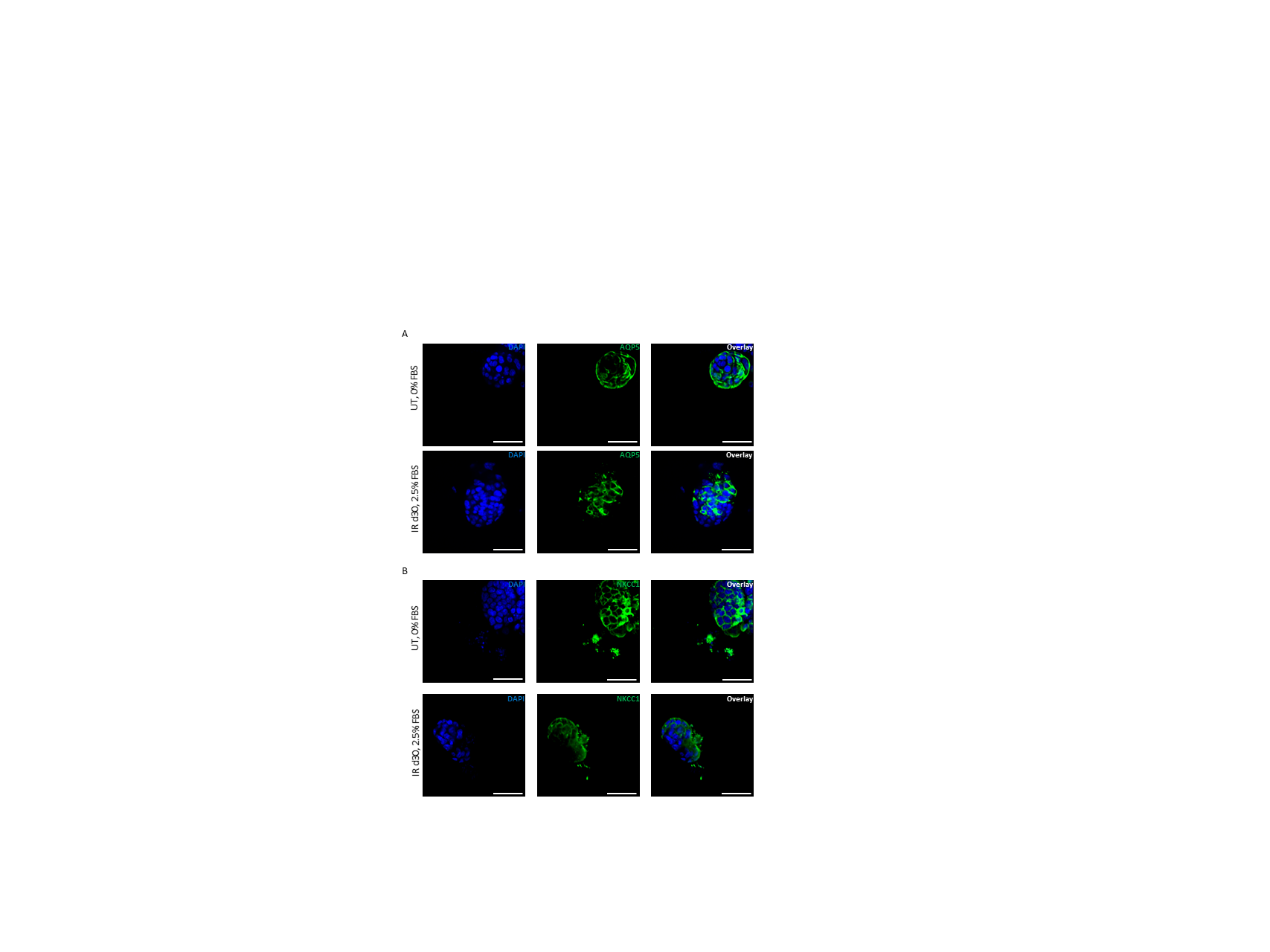

Supplement: S4 Fig — Untreated (UT) and irradiated (IR d30) parotid-derived salispheres, maintained under different FBS concentrations, were fixed after 7 days in culture and stained for Aquaporin 5 (AQP5) and NKCC1 (green). Representative confocal immunofluorescence images are shown (A-B). Scale bar = 50μm. (TIF) [file pone.0193942.s004.tif]

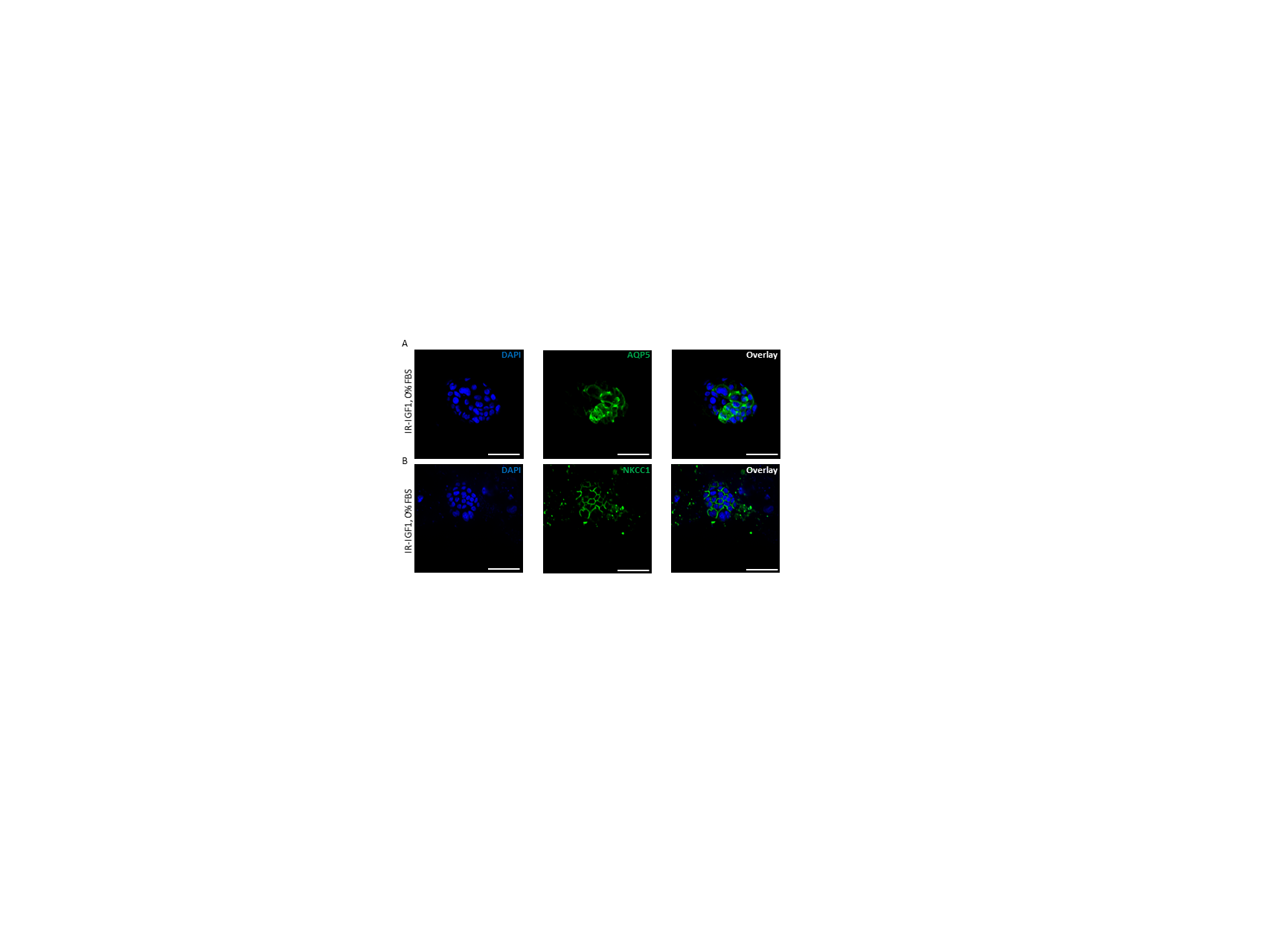

Supplement: S5 Fig — Salispheres grown from IGF1 treated parotid glands, maintained in serum free media, were fixed after 14 days in culture and stained for Aquaporin 5 (AQP5) and NKCC1 (green). Representative confocal immunofluorescence images are shown (A-B). Scale bar = 50μm. (TIF) [file pone.0193942.s005.tif]

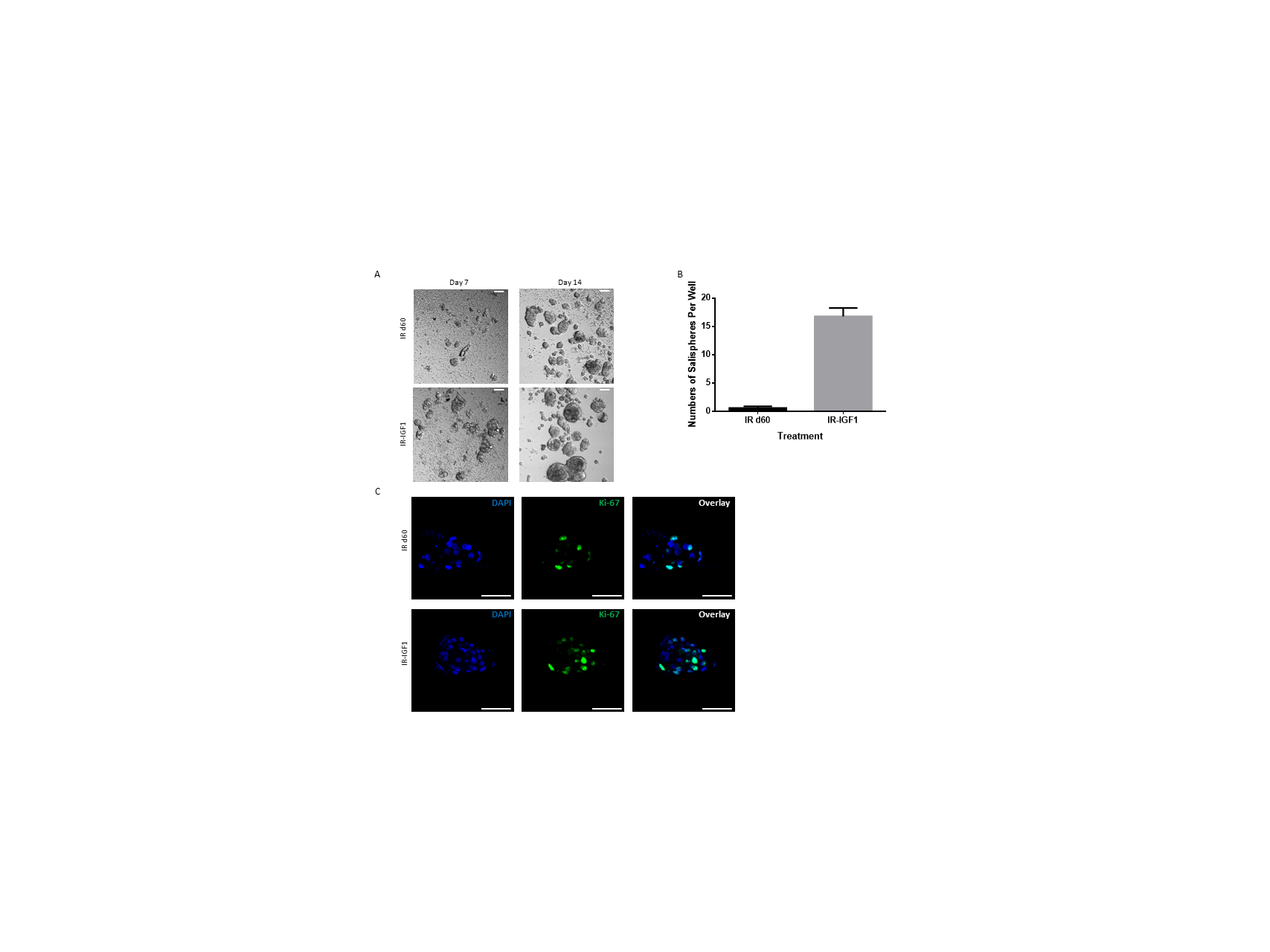

Supplement: S6 Fig — A single 5 Gy dose of radiation was given to 8 week old FVB mice followed by injections of insulin growth factor 1 (IGF1) on days 31–33 as depicted in Fig 6A. Thirty days following IGF1 treatment, parotid glands were collected for sphere formation assay. Representative bright field images of salispheres grown from irradiated (IR d60) and IGF1 treated (IR-IGF1) glands in serum-free media at different time points in culture (A). Representative graph of the average number (± SEM) of salispheres from 10 wells per treatment group on day 7 in culture from one IR d60 and IR+IGF primary sphere preparation (B). Irradiated (IR d60) and IGF1 treated (IR-IGF1) parotid-derived salispheres were fixed after 7 days in culture and stained for Ki-67 (green). Representative confocal immunofluorescence images are shown (C). Scale bar = 50μm. (TIF) [file pone.0193942.s006.tif]
